# Levothyroxine supplementation after hemithyroidectomy in patients with low-risk differentiated thyroid cancer: risk factors and withdrawal strategy

**DOI:** 10.3389/fendo.2025.1627721

**Published:** 2025-09-22

**Authors:** Jin Seok Lee, Eun Ju Ha, Ho Jung Jeong, Soo Young Kim, Hyeung Kyoo Kim

**Affiliations:** ^1^ Department of Surgery, Ajou University School of Medicine, Suwon, Republic of Korea; ^2^ Department of Radiology, Ajou University School of Medicine, Suwon, Republic of Korea

**Keywords:** thyroid carcinoma, hemithyroidectomy, levothyroxine, hypothyroidism, thyroid volumetry

## Abstract

**Background:**

The American Thyroid Association guidelines recommend maintaining thyroid-stimulating hormone (TSH) levels < 2 mIU/L postoperatively in low-risk patients. Patients with low-risk differentiated thyroid cancer, defined as intrathyroidal tumors without vascular invasion, aggressive histology, or metastasis according to ATA criteria, were included. Many patients who undergo hemithyroidectomy often maintain normal TSH levels, i.e., a euthyroid status, without taking levothyroxine after surgery. However, some patients continue to receive levothyroxine supplementation post-surgery. In this study, we analyzed the risk factors and predictors of levothyroxine withdrawal.

**Methods:**

The medical records of 132 patients who underwent hemithyroidectomy for thyroid cancer at Ajou University Hospital between February 2016 and February 2018 were reviewed. The medical records included data on demographics, type of operation, pathological findings, pre- and postoperative changes in TSH levels, levothyroxine dosage and discontinuation timing, and pre- to postoperative changes in thyroid gland volume. All patients were started on a fixed dose of levothyroxine immediately after surgery, which was subsequently tapered and withdrawn based on the TSH levels.

**Results:**

Among 132 patients who underwent hemithyroidectomy, 67 (51%) eventually withdrew from postoperative levothyroxine. Of the many dependent variables, multivariate analysis revealed the statistical significance of preoperative TSH levels *(P=0.014)*, preoperative thyroid volume measured by 3-dimensional (3D) CT, and the ratio of preoperative-to-postoperative residual thyroid volume (*P=0.026* and *P=0.012*, respectively). In the subgroup analysis of the group that resumed levothyroxine administration after levothyroxine withdrawal, only the ratio of the preoperative to postoperative residual thyroid volume was statistically significant *(P<0.043)*.

**Conclusion:**

Preoperative TSH level and thyroid volume were the most important predictors of successful postoperative levothyroxine withdrawal. The pre- to postoperative thyroid volume ratio may be affected by surgery and a ratio of <33% was significantly correlated with the ability to discontinue levothyroxine.

## 1 Introduction

Thyroid lobectomy is a widely accepted treatment for patients with benign thyroid disease and may even be curative in patients with early-stage differentiated thyroid carcinoma (DTC) (1). Theoretically, a single thyroid lobe should contain an adequate number of functioning thyrocytes to maintain a euthyroid state. Therefore, many surgeons and patients prefer unilateral thyroid resection, anticipating that postoperative thyroid hormone replacement therapy can be avoided. However, patients with preoperative suboptimal thyroid function may not retain sufficient functional thyroid tissue after lobectomy (2). Therefore, the potential need for lifelong thyroid hormone supplementation remains a critical factor in determining the extent of surgery, particularly in cases where the indications for total thyroidectomy and lobectomy are closely balanced. According to the literature, approximately 10–50% of patients require levothyroxine supplementation after thyroid lobectomy (3–7).

In cases of early DTC, a more cautious approach is warranted when considering levothyroxine therapy. As widely recognized, the 2015 ATA guidelines recommended maintaining a postoperative TSH level of <2 mIU/L in low-risk DTC patients (8). However, such targets should be individualized, taking into account the patient’s comorbidities, particularly their potential impact on bone health, cardiac rhythm, and overall quality of life. In this context, the latest ATA guidelines recommend for a risk-adapted strategy (8). Reflecting a broader shift toward treatment de-escalation—including limited surgical approaches (e.g., lobectomy), active surveillance in selected cases, and more judicious use of radioactive iodine—the guidelines emphasize the importance of carefully weighing the potential benefits of TSH-suppressive levothyroxine therapy against its potential adverse effects (9).

Recent studies have aimed to identify the preoperative predictors of postoperative thyroid hormone replacement. Nonetheless, a consensus on the standardized indications for levothyroxine supplementation has yet to be reached across institutions. Given this variability, surgeons must establish personalized criteria for postoperative thyroid hormone replacement and thoroughly inform patients during preoperative consultations about the likelihood of requiring levothyroxine supplementation following surgery. To date, several factors have been proposed as influencing the need for levothyroxine supplementation following thyroid lobectomy. These include preoperative TSH levels, the presence of microsomal antibodies, and autoimmune-related biomarkers such as thyroid peroxidase antibody (anti-TPO Ab) and thyroglobulin antibody (4, 6, 7, 10–12). However, the significance of these variables has not been consistently demonstrated across studies. Additionally, although several studies have assessed postoperative thyroid volume, studies directly correlating residual thyroid volume with the requirement for levothyroxine therapy remain limited.

This study analyzed the relationship between preoperative clinical factors and the eventual need for levothyroxine supplementation after thyroid lobectomy. Our goal was to provide predictive insights that could support clinicians in offering accurate and personalized preoperative counseling to patients.

## 2 Materials and methods

### 2.1 Study design

In this retrospective study, we reviewed the electronic medical records of 132 patients who underwent hemithyroidectomy for thyroid cancer at Ajou University Hospital between February 2016 and February 2018. To ensure minimum statistical validity, at least 50 patients per group were required for both the control and experimental groups. Considering the potential risk of dropout, a total of 132 patients were ultimately enrolled in the study. To minimize bias related to surgical technique, all procedures were performed by a single surgeon (HK). Cases of thyroidectomy after lobectomy due to contralateral lobe or lateral neck node metastasis, pregnancy within five years of lobectomy, pediatric patients under the age of 19 years, those who were lost to follow-up, and patients who were pregnant during the study period were excluded.

Patient demographics, surgical details, pathological findings, pre- and postoperative thyroid-stimulating hormone (TSH) levels, levothyroxine dosage and withdrawal timing, and pre- to postoperative changes in the volume of the thyroid gland were extracted from the patients’ medical records and reviewed retrospectively.

### 2.2 Patients and treatment

All 132 patients underwent thyroid lobectomy and were diagnosed with papillary thyroid carcinoma. After lobectomy, all patients were started on a fixed dose of levothyroxine from POD 1 (100 µg for men and 75 µg for women). All patients underwent prophylactic ipsilateral central neck node dissection and experienced no postoperative complications. All included patients underwent regular follow-up for more than 5 years at Ajou University Hospital.

### 2.3 Perioperative studies and withdrawal of thyroid hormone supplementation

Preoperative thyroid function tests (free T4 and thyroid-stimulating hormone [TSH] levels) were performed. Postoperative free T4, TSH, thyroglobulin, and thyroglobulin antibodies were measured after 2 weeks, 3 months, 6 months, and 12 months, and annually at the outpatient clinic. The total thyroid volume was measured on preoperative 3-dimensional CT (3D CT), and the residual thyroid volume was measured one year postoperatively to determine the percentage of remaining thyroid tissue.

Levothyroxine was started postoperatively with the aim of gradual reduction and eventual discontinuation, taking into account the TSH and free T4 levels and the patient’s condition. The goal of thyroid hormone supplementation is to maintain euthyroid status. Levothyroxine was discontinued if the TSH level was < 7.0 mIU/L (13, 14) and the patient was receiving a low maintenance dose of 50 µg or 25 µg, provided they agreed to discontinue the medication. If levothyroxine was discontinued during the follow-up period, TSH levels were measured three months later to determine if reinstitution was necessary.

### 2.4 Statistical analysis

Data analysis was performed using IBM SPSS statistical software (IBM Corp., Armonk, NY, USA). Fisher’s exact test or chi-square test was used to compare categorical variables. Student’s *t*-test was used to compare continuous variables, which are presented as means and standard deviations.

## 3 Results

**Figure 1:**
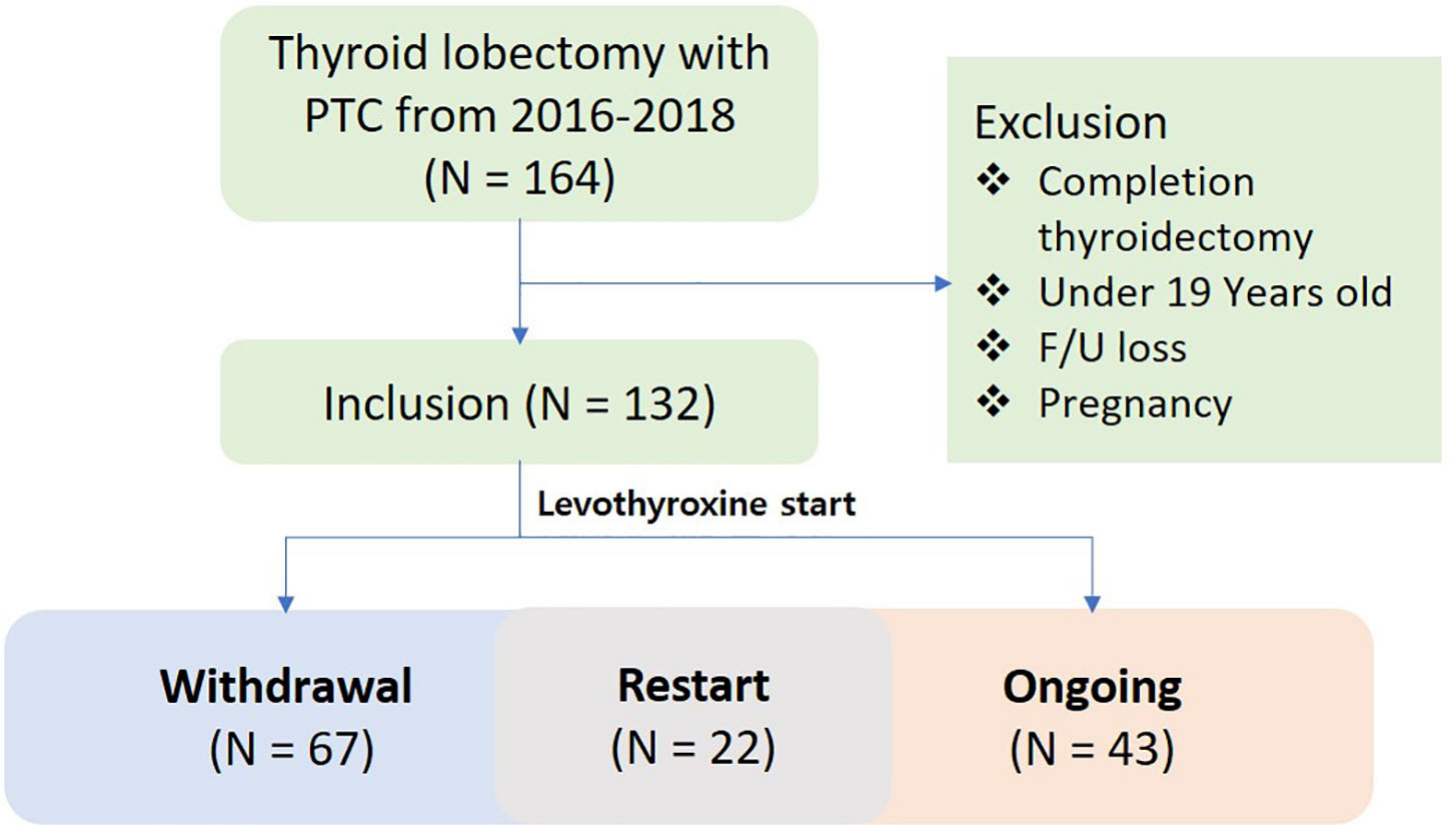
Study design. PTC, papillary thyroid carcinoma.

Data from a total of 164 patients with thyroid cancer who underwent lobectomy over a two-year period from February 2016 to February 2018 were reviewed. Of these, 132 patients were included in the final analysis after excluding those who satisfied the exclusion criteria. In the study population, 43 patients never discontinued levothyroxine, 67 patients ultimately withdrew, and 22 patients resumed levothyroxine treatment after withdrawal (**Figure 1**). The demographic data of the entire patient cohort, including sex, age, height, weight, and BMI, showed no significant differences compared to previously reported populations (5, 10, 11, 15–17). The preoperative TSH level was 1.7 mIU/L. The evaluation for thyroiditis included cases with positive preoperative serum thyroglobulin antibodies, cases suspected on ultrasound, and cases confirmed through final histopathological findings, with the numbers summarized in **Table 1**. The average preoperative thyroid volume, as measured by 3D CT, was 15.7 cm^3^, which reduced to an average postoperative volume of 5.8 cm^3^, resulting in a mean post-/preoperative volume ratio of 37.5%. The mean levothyroxine withdrawal period was 535 days (**Table 1**).

**Table 1:** Characteristics of hemithyroidectomy patients.

	Hemithyroidectomy patients (n=132)
Sex, n, (%)	
Male	32 (24)
Female	100 (76)
Age, mean ± SD, years	48.3 ± 10.5
Height, mean ± SD, cm	161.8 ± 8.7
Weight, mean ± SD, kg	65.1 ± 13.2
BMI, mean ± SD, kg/m^2^	24.8 ± 4.4
Levothyroxine, n, (%)	
Immediate post-op	132 (100)
Withdrawal	67 (51)
Preoperative TSH, mIU/L	1.7 ± 0.96
Operation site, n, (%)	
Right	73 (55)
Left	54 (41)
Isthmus	5 (4)
Contralateral nodulectomy	22 (17)
Tumor size, mean ± SD, cm	0.9 ± 0.59
Central LN metastasis, n, (%)	35 (27)
Number of metastatic LNs, mean ± SD, n	0.5 ± 1.2
Number of harvested LNs, mean ± SD, n	5.6 ± 4.1
Thyroiditis, n, (%)	
Serologic	25 (19)
US-imaging	13 (10)
pathologic	33 (25)
Volumetry, mean ± SD, cm^3^	
Preoperative	15.7 ± 6.4
Postoperative	5.8 ± 2.8
Ratio (post/pre)	0.375 ± 0.09
Mean f/u date, mean ± SD, day	2,217 ± 231
Withdrawal date, mean ± SD, day	535 ± 211
Recurrence, n, (%)	2 (1.5)

BMI, Body mass index; TSH, Thyroid stimulating hormone; LN, lymph node; US, Ultrasound; f/u, follow-up.

To analyze the risk factors or predictors in the group unable to discontinue levothyroxine, univariate and multivariate analyses were conducted, and the results were compared with those in the withdrawal group (**Table 2**). In the univariate analysis, preoperative TSH level, number of harvested lymph nodes, pre- and postoperative thyroid volume, and the ratio of post-/preoperative thyroid volume were statistically significant. Multivariate analysis confirmed that preoperative TSH, preoperative thyroid volume, and the ratio of post-/preoperative thyroid volume were statistically significant (preoperative TSH, *p=0.014*, *OR=0.581*; preoperative thyroid volume, *p=0.026*, *OR=1.469*; the ratio of post-/preoperative thyroid volume, *p=0.012*, *OR=2.539*).

**Table 2:** Univariate and multivariate analysis between levothyroxine withdrawal patients and ongoing patients.

	Withdrawal levothyroxine (n= 67)	Ongoing levothyroxine (n= 65)	P-value	
			Univariate	Multivariate
Sex, n, (%)			0.053	
Male	21 (31)	11 (17)		
Female	46 (69)	54 (83)		
Age, mean ± SD, years	47.0 ± 11.0	49.6 ± 9.9	0.149	
Height, mean ± SD, cm	163.1 ± 9.3	160.5 ± 8.0	0.096	0.427
Weight, mean ± SD, kg	65.8 ± 13.6	64.4 ± 12.7	0.533	
BMI, mean ± SD, kg/m^2^	24.7 ± 4.1	25.0 ± 4.7	0.638	
Preoperative TSH, mIU/L	1.45 ± 0.8	1.96 ± 1.0	0.002**	0.014*
Thyroiditis, n, (%)				
Serologic	11 (16)	14 (21)	0.597	
US-imaging	4 (6)	9 (14)	0.220	
Pathologic	14 (21)	19 (29)	0.366	
Operation site, n, (%)			0.260	
Right	33 (49)	40 (62)		
Left	32 (48)	22 (34)		
Isthmus	2 (3)	3 (7)		
Contralateral nodulectomy	11 (16)	11 (17)	0.938	
Tumor size, mean ± SD, cm	1.0 ± 0.7	0.8 ± 0.4	0.071	0.673
Central LN metastasis, n, (%)	23 (34)	12 (18)	0.062	
Number of metastatic LNs, mean ± SD, n	0.6 ± 1.0	0.5 ± 1.5	0.676	
Number of harvested LNs, mean ± SD, n	4.8 ± 3.7	6.4 ± 4.4	0.024*	0.063
Volumetry, mean ± SD, cm^3^				
Preoperative	17.4 ± 6.6	13.9 ± 5.6	0.001**	0.026*
Postoperative	6.8 ± 2.8	4.9 ± 2.6	0.001**	0.093
Ratio (post/pre)	0.399 ± 0.09	0.351 ± 0.10	0.004**	0.012**
Recurrence, n, (%)	1 (1.5)	1 (1.5)	0.983	

*p<0.05. **p<0.01.

BMI, Body mass index; TSH, Thyroid stimulating hormone; LN, lymph node; US, Ultrasound.

A subgroup analysis was conducted on 22 patients who required levothyroxine reinstitution after withdrawal and presented with hypothyroidism and elevated TSH levels. Among these patients, multivariate analysis revealed that only the ratio of post-/preoperative thyroid volume was statistically significant (*p=0.043*) (**Table 3**). Although the preoperative thyroid volume did not reach statistical significance (*p=0.051*), it showed a tendency toward variation. The mean TSH level at the time of levothyroxine discontinuation was approximately 2 mIU/L in both groups, with no significant difference. The period from surgery to levothyroxine discontinuation was approximately 500 days in both groups, while the average period from discontinuation to resumption was 314 days.

**Table 3:** Univariate and multivariate analysis between levothyroxine withdrawal patients and resume patients.

	Withdrawal levothyroxine (n= 67)	Resume levothyroxine (n= 22)	P-value	
			Univariate	Multivariate
Sex, n, (%)			0.074	
Male	21 (31)	2 (9)		
Female	46 (69)	20 (91)		
Age, mean ± SD, years	47.0 ± 11.0	51.9 ± 9.4	0.046*	0.521
Height, mean ± SD, cm	163.1 ± 9.3	158.1 ± 7.0	0.011*	0.248
Weight, mean ± SD, kg	65.8 ± 13.6	63.2 ± 10.0	0.395	
BMI, mean ± SD, kg/m^2^	24.7 ± 4.1	25.4 ± 4.4	0.493	
Preoperative TSH, mIU/L	1.45 ± 0.8	1.68 ± 0.9	0.287	
Thyroiditis, n, (%)				
Serologic	11 (16)	2 (9)	0.62	
US-imaging	4 (6)	2 (9)	0.806	
pathologic	14 (21)	5 (23)	0.856	
Operation site, n, (%)			0.635	
Right	33 (49)	13 (59)		
Left	32 (48)	8 (36)		
Isthmus	2 (3)	1 (5)		
Contralateral nodulectomy	11 (16)	4 (18)	0.848	
Tumor size, mean ± SD, cm	1.0 ± 0.7	0.9 ± 0.4	0.32	
Central LN metastasis, n, (%)	23 (34)	3 (14)	0.09	
Number of metastatic LNs, mean ± SD, n	0.6 ± 1.0	0.2 ± 0.5	0.062	
Number of harvested LNs, mean ± SD, n	4.8 ± 3.7	6.1 ± 4.3	0.234	
Volumetry, mean ± SD, cm^3^				
Preoperative	17.4 ± 6.6	13.1 ± 4.4	0.001**	0.051
Postoperative	6.8 ± 2.8	4.6 ± 1.9	0.001**	0.243
Ratio (post/pre)	0.399 ± 0.09	0.351 ± 0.10	0.05*	0.043*
TSH level at stop, mIU/L	2.01 ± 1.04	2.22 ± 1.27	0.435	
Levothyroxine dose at stop, µg (25–50)	50	25	0.288	
Median days to stop (min-max), days	487 (109–1470)	505 (197–984)		
Median days to resume (min-max), days		314 (76–1,437)		

*p<0.05. **p<0.01.

BMI, Body mass index; TSH, Thyroid stimulating hormone; LN, lymph node; US, Ultrasound.

Based on our findings, we suggest the following cut-off as potential predictors of successful levothyroxine withdrawal, either pre- or postoperatively. The probability of discontinuing levothyroxine was significantly higher under the following conditions: if the preoperative TSH level was less than 2.0 mIU/L (*p*<0.001) if the preoperative thyroid volume exceeds 15.3 cm^3^ (*p*<0.001), and if the ratio of post-/preoperative thyroid volume was greater than 33% (*p*=0.002).

## 4 Discussion

In the present study, we found that preoperative TSH levels, particularly below 2.0 mIU/L, were associated with an increased likelihood of levothyroxine withdrawal. In a previous study investigating the risk factors for levothyroxine use after thyroid lobectomy for benign thyroid nodules, preoperative TSH levels of ≥ 2.0 mIU/L were reported to be significantly associated with the initiation of levothyroxine, while levels of ≥ 3.0 mIU/L were significantly correlated with its continued administration (12).

These findings offer clinically relevant insights into individualized postoperative management, especially considering the variable trajectory of hypothyroidism in patients undergoing hemithyroidectomy for benign or low-risk malignant thyroid disease.

Furthermore, the same study reported that older patients were more likely to start or continue levothyroxine therapy. Another study found that preoperative TSH levels >2.5 mIU/L, as well as the presence of a positive microsomal antibody, were significantly correlated with levothyroxine supplementation (10, 18). Additionally, patients who test positive for thyroid autoimmunity-associated markers such as anti-TPO Ab or thyroglobulin antibody have a higher incidence of hypothyroidism following thyroid lobectomy (10). In the current study, we examined various modalities and markers that provide evidence of thyroiditis, including serological markers (e.g., thyroglobulin antibody), ultrasound features suggestive of diffuse thyroid disease, and thyroiditis findings on postoperative pathology. In contrast, our data suggest that patients with lower baseline TSH levels (<2.0 mIU/L)—even in the presence of equivocal markers of thyroiditis—may possess sufficient residual thyroid function to allow for successful levothyroxine withdrawal. These findings may clarify conflicting evidence regarding predictors of remnant thyroid gland function and highlight a potential threshold that could inform clinical decision-making.

Studies aimed at accurately measuring thyroid volume and postoperative residual thyroid volume have been conducted using ultrasonography and various CT modalities (16, 18–21). In the present study, 3D CT was used to measure preoperative and postoperative thyroid lobe volumes. We found that both preoperative thyroid volume and the ratio of preoperative to postoperative thyroid volume were significant predictors of levothyroxine withdrawal. Research assessing changes in the volume of the thyroid lobe after lobectomy in patients with thyroid cancer is rare and underscores the clinical significance of this study. Several previous studies have demonstrated that even when approximately half of the thyroid gland is preserved during surgery, a postoperative reduction in the remnant thyroid volume is observed (16, 22). Moreover, a study examining changes in remnant thyroid volume following surgery in patients with Graves’ disease reported that young age and a lower residual volume ratio were the two most significant factors affecting changes in remnant thyroid volume (16). Although the remnant thyroid volume or the ratio of pre- to postoperative thyroid volumes measured in our study did not provide unequivocal guidance on the optimal volume to be preserved during surgery, these findings may indirectly support the observations from studies suggesting that isthmus-preserving thyroid lobectomy is associated with a lower incidence of postoperative hypothyroidism (23, 24).

These findings are clinically relevant as they provide evidence that volumetric factors—rather than solely biochemical or serologic markers—may more accurately inform postoperative hormone requirements. Although routine use of 3D volumetry is currently limited by technical and logistical constraints, its growing integration in surgical planning workflows may enable its broader clinical application in the future.

The American Thyroid Association guidelines recommend maintaining TSH levels <2 mIU/L postoperatively in low-risk patients (8). In this study, levothyroxine withdrawal was attempted even in patients with postoperative TSH levels >2 mIU/L, provided that the patients were at a very low‐risk, i.e., showing no symptoms and maintaining normal TSH levels (in most cases ≤ 4.5 mIU/L, with only two cases <7 mIU/L). Since the publication of the 2015 ATA guidelines, ongoing advances in surgical techniques and various treatment modalities have fueled debate regarding the prognosis of patients with thyroid cancer with TSH levels >2 mIU/L, and a large-scale multicenter study is currently underway (25).

Postoperative hypothyroidism is a well-recognized concern after thyroid lobectomy. While a previous study (17) demonstrated that delaying thyroid hormone supplementation until about one-month post-surgery, in which the remnant thyroid function is evaluated, reduces the time to levothyroxine withdrawal and improves the success rate, many patients undergoing lobectomy do not receive immediate postoperative levothyroxine. In contrast, all patients included in our study were administered a predetermined dose of levothyroxine postoperatively. Despite the low incidence of immediate symptomatic hypothyroidism, this proactive approach was adopted to minimize the potential one-month period of symptoms like fatigue, cold intolerance, depression, and mood changes before treatment initiation.

Though our results are promising, this study has several limitations. First, its retrospective, single-center design may limit the generalizability of our findings. Second, the volumetric measurements required specialized 3D CT software, which may not be universally accessible. Further multicenter, prospective validation is essential to establish robust clinical thresholds and predictive models that integrate biochemical, radiological, and pathological factors.

Despite these limitations, our study highlights the importance of comprehensive preoperative assessment—including evaluation of TSH, thyroid autoimmunity, and gland volume—as a practical strategy to individualize postoperative levothyroxine therapy. If validated in larger populations, these findings could lead to improved patient counseling, enhance shared decision-making, and ultimately reduce levothyroxine overtreatment in select lobectomy patients.

## 5 Conclusion

TSH levels and thyroid volume were the most important preoperative predictors of postoperative levothyroxine withdrawal following hemithyroidectomy. Furthermore, the ratio of pre- to postoperative thyroid volume, a factor influenced by the extent of surgical resection, demonstrates that a reduction to < 33% of the original volume is significantly associated with levothyroxine withdrawal. These findings suggest that preoperative assessment of TSH and thyroid volume, along with the anticipated residual thyroid volume, can aid in predicting the likelihood of long-term levothyroxine independence.

## Data Availability

The original contributions presented in the study are included in the article/supplementary material. Further inquiries can be directed to the corresponding author.
